# *Phellinus merrillii* extracts induce apoptosis of vascular smooth muscle cells via intrinsic and extrinsic pathways

**DOI:** 10.1002/fsn3.3707

**Published:** 2023-09-19

**Authors:** Pei‐Yu Chou, Ya‐Ting Lu, Ming‐Jyh Sheu

**Affiliations:** ^1^ Department of Nursing National Chi Nan University Nantou Taiwan; ^2^ Department of Hematology & Oncology Tainan Municipal Hospital (Managed by Show Chwan Medical Care Corporation) Tainan City Taiwan; ^3^ Department of Pharmacy China Medical University, Beigang Hospital Yunlin County Taiwan; ^4^ School of Pharmacy China Medical University Taichung City Taiwan

**Keywords:** apoptosis, balloon‐injury‐induced neointima formation, Phellinus merrillii, Restenosis

## Abstract

Restenosis frequently occurs after balloon angioplasty. Percutaneous coronary intervention (PCI)‐induced artery damage is a significant part of triggering restenosis of the vascular smooth muscles (VSMC). This study aimed to study how ethanol extract of *Phellinus merrillii* (EPM) affected balloon injury‐induced overgrowth of VSMC, indicating neointima formation. Firstly, our results demonstrated that EPM notably decreased VSMC viability. A fragmentation assay and Annexin V/Propidium Iodide apoptosis assay showed that higher doses of EPM significantly induced the apoptosis of VSMC after 24 h of exposure. Total protein extracted from VSMC treated with EPM in various time and concentration periods was then conducted in Western blotting analysis. Our data demonstrated that EPM substantially elevated the p53, p21, Fas, Bax, p‐p38, and active caspase‐3 protein expressions. The results indicated that EPM induces VSMC apoptosis via intrinsic and extrinsic pathways. Also, our results demonstrated that EPM effectively attenuated the balloon injury‐induced neointima formation. In conclusion, the information offers a mechanism of EPM in inducing the VSMC apoptosis, thus as a potential interference for restenosis.

## Graphical abstract

Our results demonstrate that EPM induces the apoptosis of VSMCs by intrinsic and extrinsic apoptotic pathways. As well, we demonstrated that EPM effectively attenuated the neointima formation after balloon injury in animal studies.

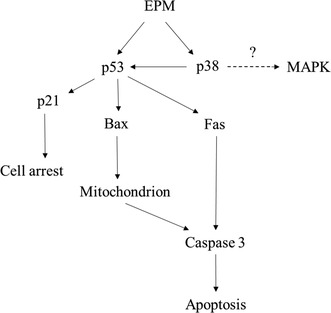

## 1 INTRODUCTION

The westernization of eating and smoking behaviors has a considerable impact, resulting in increased cardiovascular diseases (CVD), including hypertension, diabetes, hyperlipidemia, and atherosclerosis. These bring a weighty burden to the medical costs. Therefore, the prevention of CVD is a major topic that needs to be solved.

Atherosclerosis, a chronic inflammatory disorder causing the development of fibrofatty lesions in the artery tissue, is attributed to significant mortality worldwide, including peripheral artery disease, stroke, and myocardial infarction (MI) (Libby et al., 2019). In clinics, coronary angioplasty has been conducted for those who have coronary atherosclerosis (Kim et al., 2021), angina, and acute MI (AMI; Landau et al., 1994). Nevertheless, following PTCA, ~30% of the patients develop restenosis in 6 months (Serruys et al., 1988). Therefore, stents were established at that time to diminish the restenosis rate; yet, after coronary stent, restenosis happens in ~30% of the patients (Sturek & Reddy, 2002). Therefore, this results in the development of drug‐eluting stents (DES). This drug includes first‐generation (sirolimus or paclitaxel) and second‐generation (everolimus or zotarolimus), which were released from the stents. A coating of these anticancer agents on the surface of the stent contributes a lot to restenosis patients. A significant correlation has been identified between the overgrowth of VSMC and arterial restenosis after PTCA (Kibbe et al., 2000). Restenosis develops from VSMC proliferation and migration. This process originates from the media to intima layers plus the extracellular matrix formation, thus reducing vessel diameter (Labinaz et al., 1999). In this study, we intended to search for an effective strategy from natural products that could be beneficial for inhibiting neointima formation of VSMC.

Phytochemicals, specific phenolics in fruits, vegetables, and mushrooms, are recommended as the main bioactive compounds for human health advantages. Phenolics, nonessential dietary elements, have been evidenced to hinder atherosclerosis and cancer development (Martinez‐Valverde et al., 2000). Mushrooms are categorized as functional foods and an essential source of antioxidants (Arunachalam et al., 2022). Mushroom compounds are potentially treasured chemical resources for drug development and are subjected to clinical trials (Wasser, 2014). *Phellinus merrillii* (PM), called “Sanghwang in Taiwan,” is frequently used as a nutrition supplement. PM has traditionally been used for food and medicine (Chen et al., 2006). PM synthesizes several antioxidant pigments (i.e., hispolon, hispidin, and phenols) with antioxidative activity (Huang et al., 2011). Studies showed that PM exhibited antitumor activity (Huang et al., 2010; Sliva et al., 2008; Yang et al., 2017). Oxidative stress causes advanced atherosclerotic plaques, so antioxidant activity from the mushroom of our previous studies demonstrated that an ethanol extract of the PM (EPM) has been shown to increase its antioxidant activity in animals (Chang et al., 2007). Also, EPM offers hepatoprotective and antioxidant capacities in rats (Chang et al., 2007). The results support our study on the application of EPM in VSMC overgrowth. Research suggests that EPM keeps the hepatocytes from carbon tetrachloride (CCl4)‐induced liver injury (Chang et al., 2007). Moreover, *Phellinus rimosus* (Berk) Pilat has been evidenced for its antioxidative and antihepatotoxic actions (Ajith & Janardhanan, 2002). Our previous study showed that the bioactive ingredient (i.e., hispolon) from EPM significantly inhibited VSMC migration and migration without inducing apoptosis, suggesting that hispolon could block neointimal formation following balloon injury. However, the anti‐restenosis effects of EPM remain elusive.

Previous investigations have displayed that pre‐administration with antioxidants markedly diminishes the neointima formation following coronary angioplasty (Szocs et al., 2002). Study indicates that EPM containing antioxidants, including hispolon, hispidin, and inotilone (Huang et al., 2011), may be developed as a candidate to treat neointima formation in the coronary artery. In this study, our results suggested that EPM significantly suppressed the neointima formation in vivo. Moreover, the effect of EPM‐induced VSMC cell apoptosis was studied. This study was investigated to evaluate VSMC apoptosis through extrinsic (related to death receptor) and intrinsic (related to caspase cascade pathways, MAPK cascade) pathways.

## 2 MATERIALS AND METHODS

### 2.1 Materials

Chemicals including dimethyl sulfoxide (DMSO), 3‐(4,5‐dimethylthiazolyl‐2)‐2,5‐diphenyltetrazolium bromide (MTT), and others were purchased from Sigma‐Aldrich. All the reagents for the cell culture, including fetal bovine serum (FBS), penicillin/streptomycin (P/S), Dulbecco's Modified Eagle's Medium (DMEM), etc., were obtained from Gibco‐BRL Life Technologies. Hematoxylin and Eosin (H&E) staining solution was obtained from Merck. The antibody against p53 (#sc‐126), p21 (#sc‐166630), Fas (#sc‐74540), Bax (#sc‐7480), p‐p38 (#sc‐7973), caspase‐3 (#sc‐56053), mouse anti‐rabbit IgG‐HRP (#sc‐2357), and goat anti‐mouse IgG‐HRP secondary antibody (#sc‐2354) were obtained from Santa Cruz Biotechnology, Inc. The PM was obtained from the Ji Pin mushroom store in Nantou, Taiwan. The PM was identified by Professor Yu‐Cheng Dai (Institute of Applied Ecology, Chinese Academy of Science).

### 2.2 Cell line and cultures

A10, an embryonic thoracic aorta smooth muscle of a rat acquired from the Bioresource Collection and Research, was kept in DMEM, supplemented with FBS (10%), and P/S (1%) at 37°C in a humidified atmosphere filled with 95% air, and 5% CO_2_. A10 cells cultured in DMEM containing 0.5% FBS and 15% FBS served as a negative and positive control, respectively.

### 2.3 Extract of *Phellinus merrillii*

According to our previous report, PM (1.5 kg) was carefully soaked with 70% of ethyl alcohol (5 L) for active compound extraction at room temperature (~25°C). Firstly, the solution was filtered while the residue was extracted thrice. The collected filtrates were mixed and evaporated by a vacuum to obtain the crude extracts (60 g) (Chien et al., 2012).

### 2.4 Cytotoxicity assay

Cell cytotoxicity analysis was conducted by the MTT assay. A10 was seeded in 96‐well plates (1 × 10^4^ cells each well) in DMEM containing 15% FBS for 24 h. After 24 h, the cell was washed with PBS and then exposed to either 15% FBS only or various dilutions (10, 20, 40, and 80 μg/mL) of EPM. After 24 h, 10 μL of the MTT was pipette into each well (0.5 mg/mL was the end‐point concentration) and was incubated at 37°C for 2 h. Finally, the purple formazan crystals produced by the viable cells from the MTT presented were measured at the absorbance length (570 nm) by an ELISA reader (Bio‐Tek).

### 2.5 Cell death assays

Cell apoptosis was evaluated by Annexin‐V apoptosis detection kit with propidium iodide (PI) (#ab14085; Abcam) by the FACS Calibur™ system. A10 was administered with various doses of EPM (10, 20, 40, and 80 μg/mL) in six‐well plates for 24 h. Briefly, washing twice with PBS, 400 μL 1× annexin‐V binding buffer was added to each tube. Then, A10 was stained with annexin‐V fluorescein isothiocyanate (5 μL) and PI buffer (10 μL) per tube. A 5 min incubation at RT in the dark, the fluorescent intensities of each test were measured with a FACSCanto flow cytometer (BD Biosciences) and fluorescence microscope.

### 2.6 DNA fragmentation assay

After the centrifugation at 150 *g* for 5 min, A10 (1 × 10^5^ cells) were collected and then washed with PBS buffer. Cells were suspended in Tris buffer (10 mM; pH 7.4), ethylenediaminetetraacetic acids (10 mM, EDTA; pH 8.0), 0.5% TritonX‐100 and saved at 4°C for 10 min. After that, the supernatant was incubated with 2 mL of RNase A (20 mg/mL) and 2 mL of proteinase K (20 mg/mL) at 37°C for 1 h and then maintained in 120 μL of isopropanol at −20°C overnight. After, the supernatant was centrifuged at 15,000 *g* for another 15 min. DNA was dissolved in TE buffer (2‐amino‐2‐hydroxymethyl‐propane‐1,3‐diol–EDTA), 20 μg genomic DNA was loaded onto 2% agarose gel electrophoresis at 50 V for 40 min, stained with ethidium bromide, visualized under UV light, and photographed.

### 2.7 Western blotting analysis

A10 were lysed in RIPA lysis buffer (Thermo Fisher Scientific Inc.) with phosphatase and protease inhibitor cocktails (Sigma‐Aldrich). Concentrations of the protein were assayed by a Pierce BCA Protein Assay Kit (Thermo Fisher Scientific Inc.). 20 μg of total protein samples were separated by 10% SDS‐PAGE and transferred to PVDF membranes. And membranes were blocked with nonfat dry milk (5%) in phosphate‐buffered saline (PBS)–Tween for 1 h. and then probed with the antibodies (anti‐p‐p38 phosphorylation, anti‐p53, anti‐p21, anti‐FAK, anti‐Bax, anti‐Fas, and anti‐cleaved caspase 3) overnight at 4°C. The blots were then incubated with the goat anti‐mouse IgG secondary antibody for another hour, followed by development with the electrochemical luminescence reagent and exposure to Hyperfilm. Protein levels were detected by the Image Quant system (LAS 4000; GE Healthcare) with auto settings (sensitivity: normal; exposure time: auto expose). To establish the normalization of the band intensity of the protein is to the band intensity of the internal control protein (α‐tubulin), and the expression level of the protein is expressed as fold change relative to the value of the control group.

### 2.8 Balloon angioplasty

Male Sprague–Dawley rats (SD rats; 200–250 g) were bought from BioLASCO and randomly divided into three groups (three rats per group). The animals housed in a 12‐h light–dark cycle were given ad libitum access to food and water. Balloon injury of this carotid artery was executed by a balloon embolectomy catheter (2F × 80 cm; Biosensors International Technology) in the left common carotid artery (Wu et al., 2004). The three groups of the SD rats included the balloon‐injured group, and two doses of EPM (40 and 80 mg/kg) were administered daily for two continuous weeks before balloon injury through gastric intubation. At 2 weeks after balloon injury, rats were sacrificed by carbon dioxide overdose. Animals were perfused with 0.9% normal saline till free of blood and 10% formalin perfusion fixation for histological examination. Both sides of common carotid arteries were collected, embedded in paraffin, crosscut into 10 μm thick sections, and then stained by the H&E staining solution (Merck). The areas of the carotid arterial wall's lumen, media, and neointima layers were measured with Image J 1.44 software (National Institutes of Health). The Institutional Animal Care and Use Committee (IACUC) ethics committee of China Medical University approved all experimental processes involving these rats. This study was approved by the IACUC (animal review number: 2005‐40).

### 2.9 Statistical analysis

Data are stated as means ± standard deviation (SD). A one‐way analysis of variance (ANOVA) combined with Dunnett's test was used to determine the significance between multiple groups. A *p*‐value < .05 was considered statistically significant.

## 3 RESULTS

### 3.1 Extract of *Phellinus merrillii* on cell viability of VSMC

**FIGURE 1:**
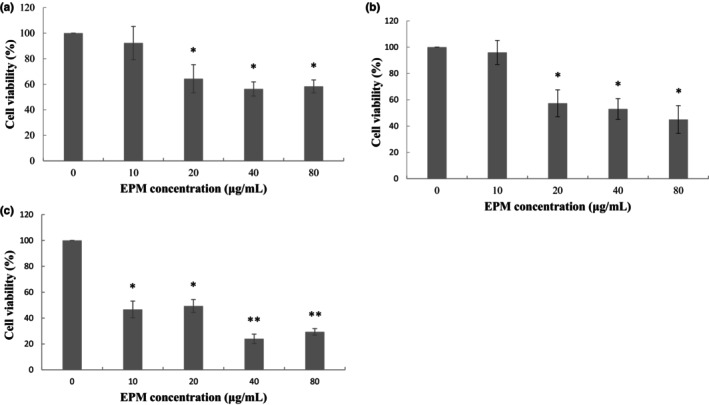
Extract of *Phellinus merrillii* (EPM) decreased the VSMC viability detected by MTT assay. The cells were incubated for 24 (a), 48 (b), and 72 h (c) with 15% FBS alone (control) or with various concentrations of EPM (10, 20, 40, and 80 μg/mL). Values were the means of three independent experiments, with standard errors represented by vertical bars. The mean value significantly differs from the control group: *p < .05; **p < .01.

As the overgrowth of VSMC plays a significant role in restenosis, we determined the inhibitory effects of EPM on VSMC by MTT assay. Our results showed that EPM significantly reduced VSMC viability in a dose‐ and time‐dependent manner (A). EPM (20, 40, and 80 μg/mL) effectively inhibited VSMC viability at 36%, 44%, and 42%, respectively (*p* < .01) after 24 h incubation period (Figure 1a). Following 48 h incubation, EPM (20, 40, and 80 μg/mL) effectively inhibited VSMC viability at 43%, 46%, and 55%, respectively (*p* < .01; Figure 1b). After 72 hr. incubation, EPM (10, 20, 40, and 80 μg/mL) significantly inhibited VSMC viability at 53%, 51%, 76%, and 71%, respectively (*p* < .01; Figure 1c).

### 3.2 Extract of *Phellinus merrillii* induced VSMC apoptosis

**FIGURE 2:**
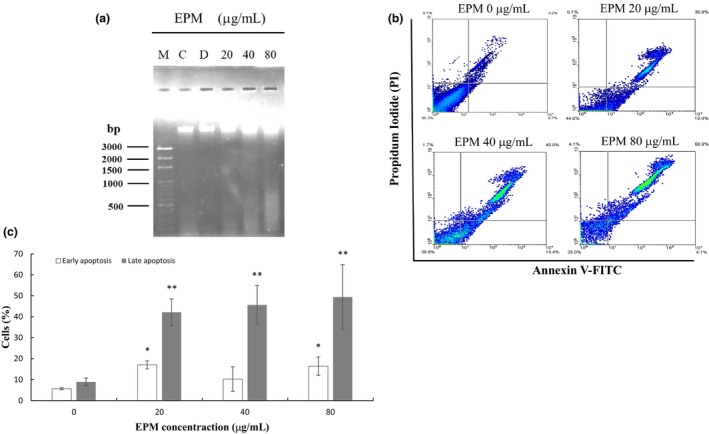
An extract of *Phellinus merrillii* (EPM) induced VSMC apoptosis. (a) DNA fragmentation assay indicated that an extract of *Phellinus merrillii* (EPM) induced VSMC apoptosis. A10 were cultured in 15% FBS alone or plus with EPM (20, 40, and 80 μg/mL) for 48 h. M, marker; C, control, 15% FBS; D, A10 cells treated with dimethyl sulfoxide (DMSO) as vehicle control. (b) Membrane translocation of phosphatidylserine (PS) was determined by staining with Annexin V‐FITC/propidium iodide (PI) and then analyzed by flow cytometry in VSMC treated with 20, 40, and 80 μg/mL for 48 h. (c) The figure shows the results of the statistical analysis. Three independent experiments measured the mean value. *p < .05; **p < .01. when compared with the control group (EPM 0 μg/mL).

**FIGURE 3:**
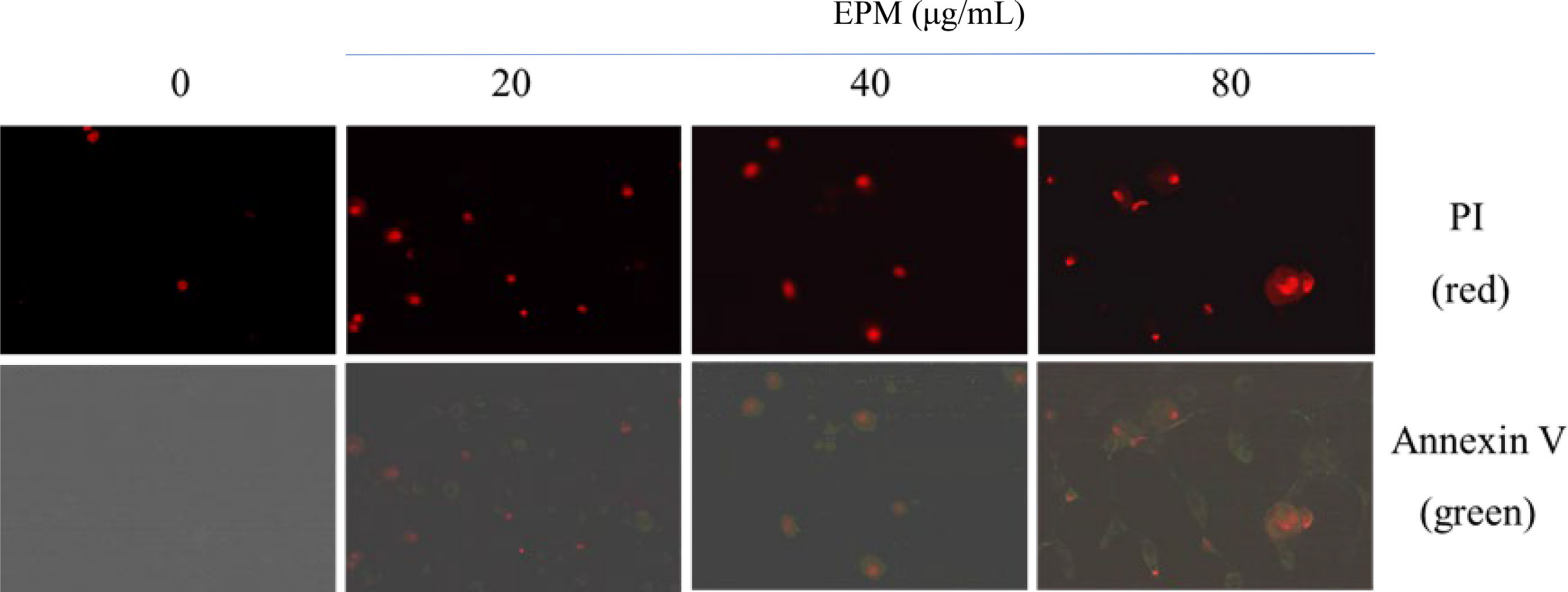
Annexin V/Propidium Iodide apoptosis assay showed that EPM (20, 40, and 80 μg/mL) significantly induced VSMC cell apoptosis after 48 h of exposure, examined by fluorescence microscopy.

We assumed that the cytotoxic effects of EPM might be regulated by apoptosis. Our results showed that after exposure of 48 h. EPM (80 μg/mL) induced DNA fragmentation in VSMC. The results indicate that EPM only at a higher dose (80 μg/mL) induced VSMC apoptosis, but lower doses of EPM did not cause VSMC apoptosis (Figure 2a). Data from the Annexin V/Propidium Iodide apoptosis assay, EPM (20, 40, and 80 μg/mL) significantly induced VSMC cells apoptosis at the same time interval of exposure (i.e., 48 h), examined by flow cytometer (Figure 2b,c) and fluorescence microscopy (Figure 3). These results showed that the EPM induced VSMC apoptosis.

### 3.3 Effects of extract of *Phellinus merrillii* on p53, p21, Fas, Bax, p‐p38, and active caspase‐3 protein expressions

**FIGURE 4:**
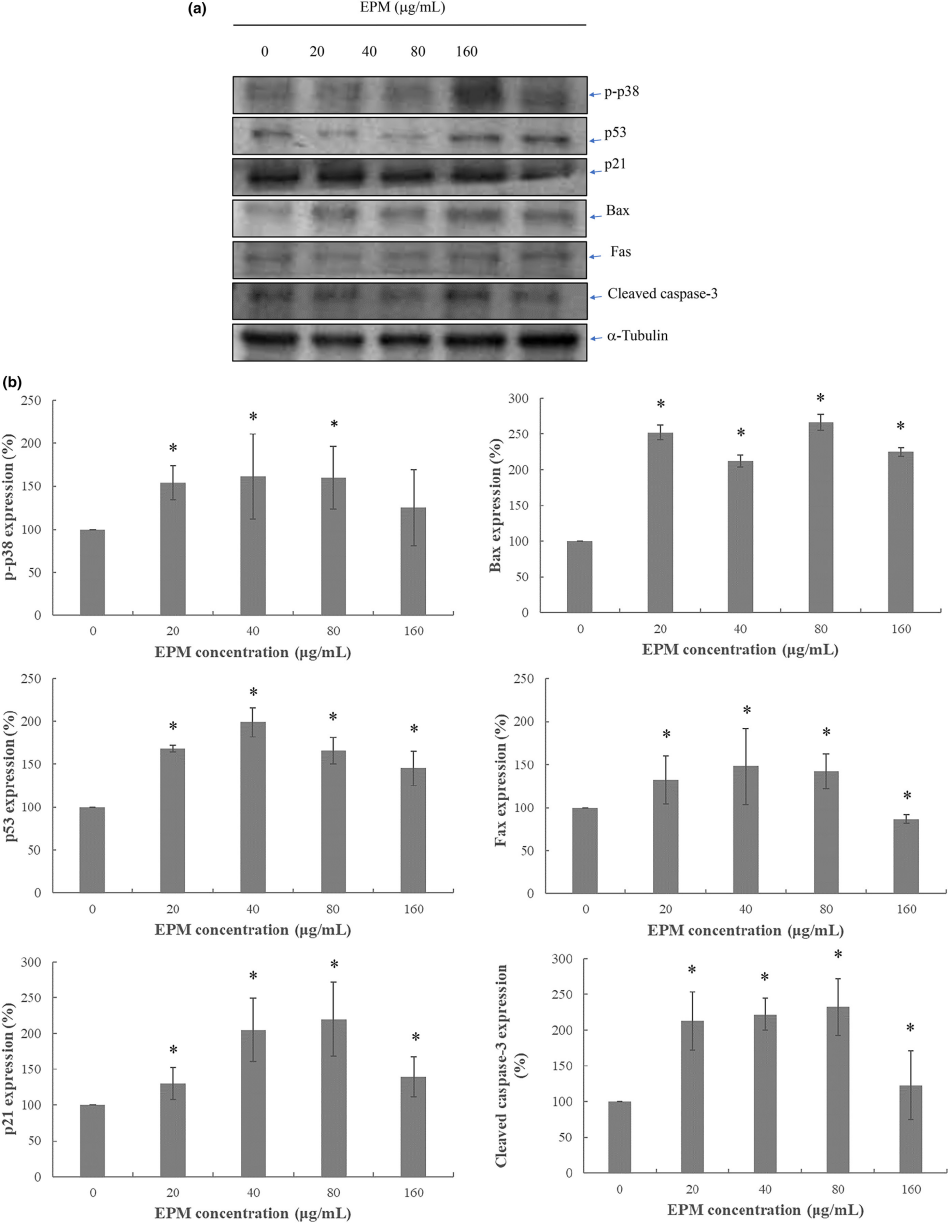
Effects of extract of EPM on protein levels of p53, p21, Fas, Bax, and p‐p38. Also, active caspase‐3 protein expression levels in VSMC. (a) The cells were treated with several different concentrations of EPM (20, 40, 80, and 160 μg/mL) for 24 h. Cells getting 15% FBS were the positive controls (0). The values indicated the density proportion of each protein compared with the control. A typical immunoblot from three independent experiments with similar results was shown. (b) The figure shows the results of the statistical analysis. The mean value was significantly different from that of the control group: *p < .05.

The 15% FBS‐stimulated VSMC were treated with EPM at 20, 40, and 80 μg/mL for 24 h, respectively. Total proteins were extracted from EPM‐treated VSMC and evaluated by Western blotting assay. To explore the modulation of apoptosis through both extrinsic and intrinsic pathways by EPM in VSMC, we determined p‐p38, p53, p21, and proteins associated with apoptosis expression levels. As shown in Figure 4a, our results showed that EPM at various concentrations significantly increased p53, p21, Bax, Fas, p38, and active caspase‐3 at 20, 40, 80, and 160 mg/kg. (Figure 4a,b).

### 3.4 Extract of *Phellinus merrillii* on balloon injury‐induced neointimal formation on the carotid artery

**FIGURE 5:**
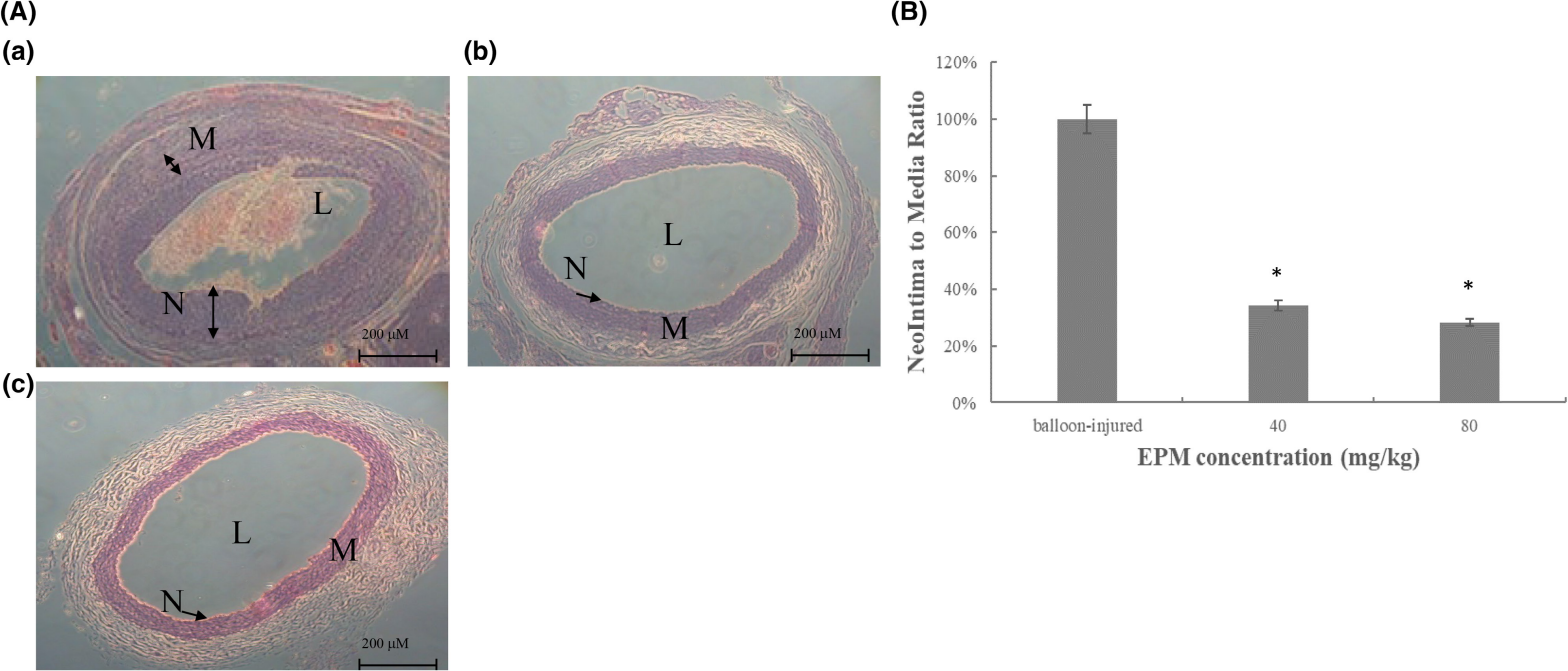
Responses of rat carotid arteries to balloon injury and the effects of an extract of EPM on balloon injury. The left panel represented the low‐power (100×) observations from a balloon‐injured vessel (a), a balloon‐injured vessel treated with EPM at 40 μg/mL (b), and a balloon‐injured vessel treated with EPM at 80 μg/mL (c). L, lumen; M, media; N, neointima.

To examine the effectiveness of EPM in preventing neointimal formation, the animals were treated with EPM (40 and 80 mg/kg) for 2 weeks before balloon injury. After 14 days of EPM (40 and 80 mg/kg) treatment, these arteries were harvested, and the histological study assayed the neointimal formation. Using image analysis, area ratios of neointimal and media layers were calculated, and reductions in the area ratio of 40 and 80 mg/kg treated groups, respectively (Figure 5A(b,c)). Our results showed that EPM at higher doses (40 and 80 mg/kg) meaningfully suppressed the VSMC outgrowth (Figure 5A,B).

## 4 DISCUSSION

CVD is the chief cause of mortality globally. Several factors, including oxidative stress and inflammation, lead to the development of CVD; consequently, antiinflammatory and antioxidant agents become the potential therapeutic targets of CVD (Pereira et al., 2021). Significantly, numerous basic and clinical studies have described the antioxidative characteristics of nutrition diets for CVD (Aune, 2019; Burr et al., 1989; Maleki et al., 2019; Taleb et al., 2018; Wu et al., 2004). Previous research indicated that an active ingredient from mushrooms has been revealed to obstruct VSMC proliferation after balloon injury (Yoo et al., 2012). However, the mushroom ethanol extract has not shown its effects on PTCA‐related restenosis. Based on previous research, EPM demonstrated its antioxidant activity in animals (Chang et al., 2007). Therefore, we hypothesized that EPM could have a role in inhibiting neointima formation. In this research, we first demonstrated that EPM effectively attenuated the neointima formation after balloon injury (Figure 5). Besides, our result showed that EPM caused VSMC apoptosis through the evidence of DNA fragmentation (Figure 2a). The molecular mechanism showed that EPM‐induced VSMC apoptosis by intervening with intrinsic and extrinsic apoptotic pathways (Figure 4).

Neointimal formation indicates the pathological conditions (i.e., atherosclerosis) because of the proliferation and migration of VSMC in the layer of the tunica intima, leading to the narrowed lumen of the artery (i.e., coronary or carotid), which may cause the insufficiency of vascular symptoms (Christen et al., 2001). The arterial VSMC restenosis plays a critical role that limits the achievement of the PTCA treatment. Both the proliferation and migration of VSMC are attributed to neointima formation following balloon injury. Consequently, modulation of the VSMC overgrowth provides a promising therapeutic suggestion (Ross, 1999). DES was applied in clinics to overcome the problem. These drugs include first‐generation (sirolimus or paclitaxel) and second‐generation (everolimus or zotarolimus) medications. Studies have revealed that sirolimus and like analogs (i.e. everolimus, zotarolimus, sirolimus, or novolimus) confirmed the prospective inhibitory impacts in neointimal formation (Cremers et al., 2012; Lee & de la Torre Hernandez, 2018; Semsroth et al., 2009; Suzuki et al., 2001); however, myocardial infarction and thrombosis could develop. We hypothesized that certain natural products with antioxidative activity may produce their drug‐eluting effects but have fewer adverse effects. Our study first verified that EPM employed an effective inhibitory impact on the development of VSMC (Figure 1). Like the sirolimus, EPM showed its apoptotic effects on VSMC (Figures 2 and 3). Afterward, the results suggest that EPM induces VSMC apoptosis through extrinsic and intrinsic pathways (Figure 4). Our results showed that EPM caused a substantial decrease in neointimal formation 14 days after carotid arterial injury in animals (Figure 5).

Several intracellular stimuli recognized by different proteins direct these signals to the mitochondria and increase its membrane permeability (Chipuk et al., 2006), which is regulated by B‐cell lymphoma 2 protein (Bcl‐2) family (i.e., Bax and Bak). Bax and Bak are produced in higher eukaryotes that can penetrate the outer membrane of the mitochondria and facilitate cell apoptosis (Chipuk et al., 2004). The family of Bcl‐2 proteins includes 25 pro‐apoptotic and anti‐apoptotic members. The health of the cells is determined by the balance between the anti‐apoptotic (i.e., Bcl‐2 and Bcl‐xL) and pro‐apoptotic proteins (i.e., Bax and Bak; Czabotar et al., 2014). The pro‐apoptotic members such as Bax, which stimulate and insert into the outer cell membrane of the mitochondrial, lead to the release of cytochrome c (Chipuk et al., 2006). Consequently, cytochrome c interacts with apoptosis protease‐activating factor 1 (Apaf1) and forms a complex known as the apoptosome in the cytosol (Zou et al., 1997). After that, apoptosome activates caspase‐9 (initiator caspase), then caspase‐3 (execution caspase). Finally, this cascade causes cell apoptosis (Jin & El‐Deiry, 2005). p53, a tumor suppressor gene, employs the anticancer action by apoptosis (Soussi, 2000). p53, a tumor suppressor gene, mainly contributed to inducing cell death (i.e., apoptosis) by transcription‐dependent and ‐independent processes. Also, nuclear p53 transcriptionally activates many pro‐apoptotic BCL‐2 family genes (i.e., Bax); however, it inactivates the expression of anti‐apoptotic genes, such as Bcl‐2, causing mitochondrial apoptosis. p53 is evident to activate the pro‐apoptotic Bax protein, increase the mitochondria permeability, and engage the apoptotic program. The accumulation of p53 in the cytosol acts to the third Bcl‐2 homology domain (BH3)‐only subset of pro‐apoptotic Bcl‐2 proteins to stimulate Bax and initiate apoptosis (Chipuk et al., 2004). Besides, p21 is a cell‐cycle inhibitor that leads to cell arrest in G1/S and G2/M transitions by reducing cyclin‐D and cyclin‐E, respectively (Zaldua et al., 2016). Our results showed that EPM significantly enhances the protein expressions, including p53, p21, Bax, and caspase‐3 (Figure 4). This is consistent with the hypothesis that EPM induces VSMC apoptosis by the mitochondrial pathway.

The extrinsic pathway of apoptotic signaling starts with the binding of various ligands (i.e., tumor necrosis factor (TNF), Fas ligand (Fas‐L), and TNF‐related apoptosis‐inducing ligand (TRAIL)) to the death receptor (i.e., TNFα1, Fas, and TRAIL receptors; Guicciardi & Gores, 2009; Kashyap et al., 2021). When the ligand binds with its specific death receptor, these receptors bind to adapter proteins, including the Fas‐associated death domain (FADD) and TNF receptor‐associated death domain (TRADD). This cascade is triggered by the active caspase‐8, which brings about cell death by damage to the nucleus (Jin & El‐Deiry, 2005). Like the sirolimus and analogs, EPM showed its apoptotic effects on VSMC through the extrinsic pathway (Figures 2 and 3). It is well‐recognized that tumor necrosis factor‐α (TNF α) causes a mild response of the p38‐ mitogen‐activated protein kinases (MAPKs), bringing about solid activation of the stress‐related the c‐Jun NH(2)‐terminal kinases (JNKs) (Kant et al., 2011). When binding to TNF receptor type 1, TNF causes the activation of caspase‐8, thus inducing apoptosis (Gaur & Aggarwal, 2003). When the Fas ligand (FasL) binds to the Fas receptor (a death receptor) on the cell surface, it induces cell apoptosis (Wajant, 2002). Our results also showed that administered EPM significantly increased FAS and caspase‐3 protein expression in VSMC. These results suggest that EPM may cause VSMC apoptosis by the extrinsic apoptotic pathway. Redox homeostasis is critical for normal cellular function (Kamata & Hirata, 1999; Torres & Forman, 2003). Normal cellular metabolism produces low reactive oxygen species (ROS) and nitrogen species (Rahman et al., 2023). However, increased production can lead to oxidative stress, cardiovascular disease, and cancer (Dey et al., 2022; Dubois‐Deruy et al., 2020). Many studies have shown that increased ROS are essential mediators of proliferation or apoptosis and lead to the activation of various signaling molecules and pathways, including MAP kinase (Yang et al., 2022). EPM also markedly enhances the protein expression of p‐p38, Fas, and caspase‐3 protein levels in VSMC (Figure 4).

**FIGURE 6:**
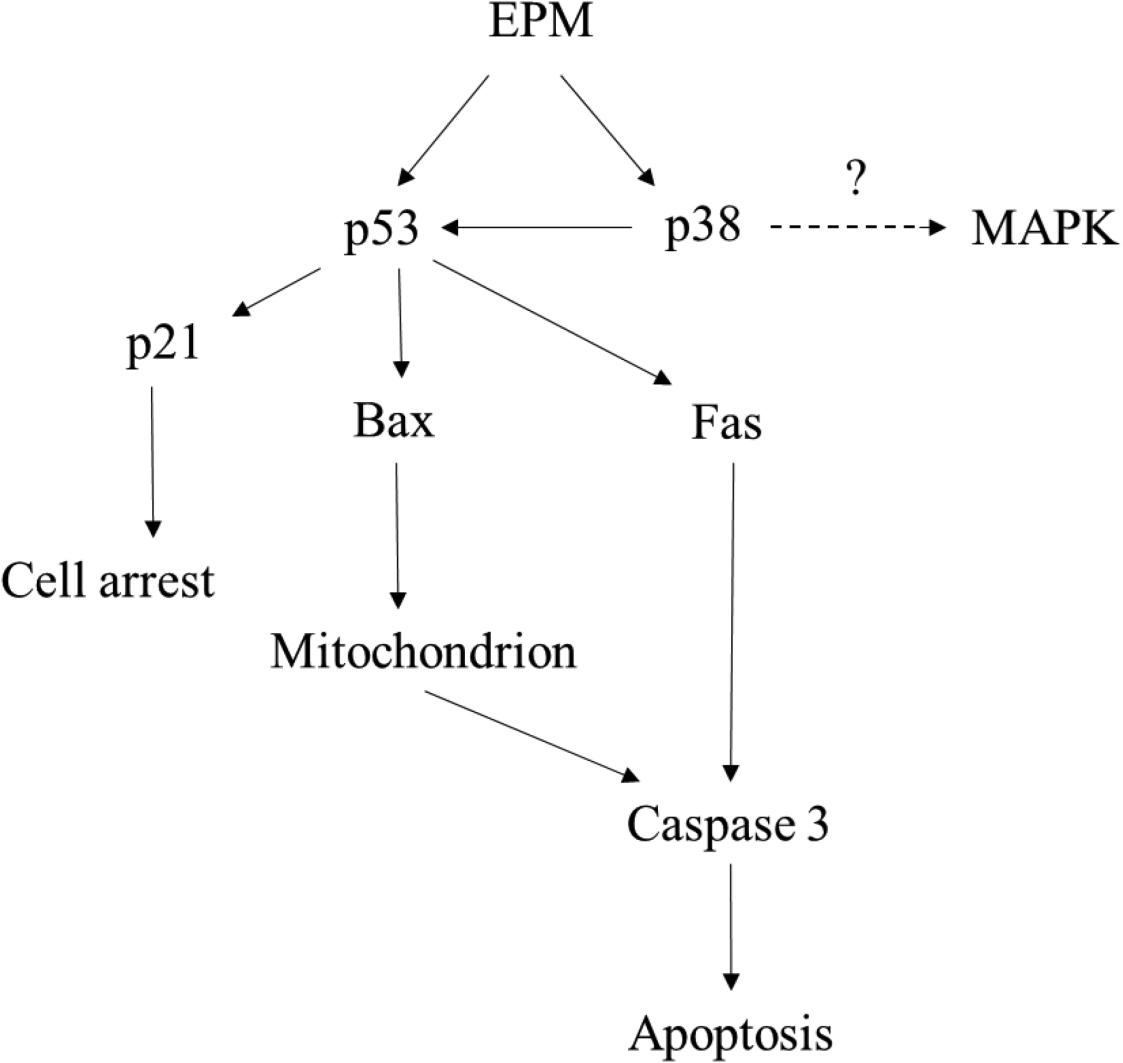
Schematic overview of the pharmacological mechanisms of EPM in preventing restenosis in VSMC. MAPK, mitogen‐activated protein kinase; p53, tumor protein p53; p21, also known as cyclin‐dependent kinase inhibitor 1; p38, p38 mitogen‐activated protein kinases.

In conclusion, we first demonstrated that EPM‐induced VSMC apoptosis through extrinsic (related to death receptor; Fas) and intrinsic (related to caspase cascade pathways; caspase‐3 and MAPK cascade; p38) pathways. These inhibitory processes may partially control VSMC apoptosis and balloon angioplasty‐induced neointimal formation (Figure 6). Our results showed that EPM, a mushroom, may possess possible applications in preventing restenosis.

## AUTHOR CONTRIBUTIONS

**Pei‐Yu Chou:** Data curation (equal); formal analysis (equal); investigation (equal); methodology (equal); writing – original draft (supporting). **Ya‐Ting Lu:** Data curation (equal); formal analysis (equal); investigation (equal); methodology (equal); writing – original draft (equal). **Ming‐Jyh Sheu:** Conceptualization (lead); formal analysis (equal); investigation (equal); project administration (lead); supervision (lead); validation (equal); writing – original draft (lead); writing – review and editing (lead).

## FUNDING INFORMATION

This study was sponsored by NSC‐94‐2320‐B‐039‐022 from the National Science Council, Taiwan.

## CONFLICT OF INTEREST STATEMENT

The authors declare that they have no conflict.

## Data Availability

The data supporting this study's findings are available on request from the corresponding author. The data are not publicly available due to privacy or ethical restrictions.
